# Joint Video Super-Resolution and Frame Interpolation via Permutation Invariance

**DOI:** 10.3390/s23052529

**Published:** 2023-02-24

**Authors:** Jinsoo Choi, Tae-Hyun Oh

**Affiliations:** 1 Department of Electrical Engineering, KAIST, Daejeon 34141, Republic of Korea; 2 Department of Electrical Engineering and Graduate School of AI, Pohang University of Science and Technology (POSTECH), Pohang 37673, Republic of Korea; 3 Department of Artificial Intelligence, Yonsei University, Seoul 03722, Republic of Korea

**Keywords:** video enhancement, super-resolution, frame-rate up-conversion

## Abstract

We propose a joint super resolution (SR) and frame interpolation framework that can perform both spatial and temporal super resolution. We identify performance variation according to permutation of inputs in video super-resolution and video frame interpolation. We postulate that favorable features extracted from multiple frames should be consistent regardless of input order if the features are optimally complementary for respective frames. With this motivation, we propose a permutation invariant deep architecture that makes use of the multi-frame SR principles by virtue of our order (permutation) invariant network. Specifically, given two adjacent frames, our model employs a permutation invariant convolutional neural network module to extract “complementary” feature representations facilitating both the SR and temporal interpolation tasks. We demonstrate the effectiveness of our end-to-end joint method against various combinations of the competing SR and frame interpolation methods on challenging video datasets, and thereby we verify our hypothesis.

## 1. Introduction

Recent super-resolution (SR) approaches [1,2,3,4,5] and frame interpolation methods [6,7,8] have shown promising results demonstrating significant advancements in their respective areas. Those methods can enhance the camera sensors’ physical limitations to go beyond only with software-level algorithms. However, SR and frame interpolation have been mostly regarded as separate research topics. To achieve both goals of SR and frame interpolation, a naive solution would be to sequentially apply them to the given video frames. This is sub-optimal since each task is done independently without any complementary interaction.

In this work, we propose a joint SR and frame interpolation method to perform spatio-temporal SR, where it conducts temporal frame generation as well as higher spatial resolution in a joint manner, applicable to tasks including spatio-temporal video compression and enhancement. Our work is built upon the hypothesis that reconstruction of higher resolution images and inter-frame recovery are both heavily influenced by the input texture information but in slightly different aspects. The frame interpolation task essentially makes use of neighboring frames to extract texture motion for middle-frame synthesis. Similarly, this concept is the underlying key idea of the multiple image SR task which takes advantage of the different aliasing from each frame. While both tasks leverage information with significant overlap, the features learned in each task would convey different characteristics. Thus, sharing the texture information while learning the spatial and temporal aspects as multi-task learning would benefit both tasks by preventing loss of a chance to learn complementary information.

We coin the learned features as *complementary features*, since the given different frames provide complementary information, which in turn provides complementary interaction between the SR and frame interpolation tasks. Given candidates of texture features from the frames, to extract a feature representation containing the complementary knowledge across the two frames, we need an information aggregation mechanism. A typical way of aggregating such temporal information would be convolutional neural networks (CNN) with frames concatenated along the channel dimension or recurrent architectures, but these may introduce asymmetric influence on the set of inputs [9,10,11]. To equally consider the feature candidates i.e., agnostic to input order, we argue that an order (permutation) invariant operation [10] is necessary for multi-frame based video processing. We extend recent ideas on permutation invariant convolutional neural networks with residual connections and the attention mechanism to construct effective representation, also capable of dealing with occlusion and disocclusion between frames.

In summary, our work has the following contributions:

- We propose the permutation invariant residual block (PIRB) which can process the input frames in a permutation invariant manner while effectively extracting the complementary features. Thereby, we demonstrate the visually pleasing quality. In turn, the learned features effectively shepherd both tasks.
- We propose the feature attention mechanism for the proposed task to effectively focus on important regions and handle unwanted artifacts.

We evaluate our method on multiple datasets including the Vimeo90k [12], Vid4 [13], and SPMCS [14] against various combinations of top performing state-of-the-art SR and frame interpolation methods. Our approach demonstrates superior performance in terms of quantitative comparisons and visual results.

## 2. Related Work

### 2.1. Super Resolution

The main goal of super resolution is to enhance the spatial resolution of an image or video. Single image super resolution (SISR) is a sub-branch within the SR category which deals with single image inputs. Since only one image is given, SISR is the most ill-posed task among the SR categories, i.e., compared to multiple image super resolution (MISR). Thus, most SISR approaches take a data-driven texture synthesis approach to explicitly learn the mapping distribution from LR to HR images. For the SISR task, Ref. [15] first proposed the deep CNN approach, pioneering the deep learning approaches to SR. Notable deep architectures have followed this work incorporating sub-pixel CNN [16], residual networks [3,17], recursive CNN [18], dense connections [5], channel attention [19]. Recently, Haris et al. [1] proposed the deep back-projection network by projecting upsampled and downsampled features in a densely connected way.

Video super resolution (VSR), also referred to as MISR, aims to reproduce the *true* HR image by making use of neighboring frames. Early works include [20] which first incorporated deep learning via warped frames. Following this work, VSR has been approached by a similar explicit alignment approach: alignment-based [21], sub-pixel motion compensation [14], feature level motion alignment [22], and joint training of optical flow for specific tasks [12]. As another category, ref. [23] proposed the first end-to-end VSR method, and the subsequent works have proposed by pure-inference without matching (e.g., temporal adaptive network [24], 3D CNN [25,26]) or implicit alignment (e.g., recurrent back-projection network [2], spatio-temporal attention module [4], burst imaging [27]). While these works focus on spatial SR based on motion estimation, our work deals with both SR and temporal interpolation. Among this line of work, we are the first to tackle the order invariance property.

### 2.2. Frame Interpolation

Video frame interpolation is a task of generating an intermediate frame given neighboring frames. For one of the earlier works, Niklaus et al. [8,28] proposed to take two image patches and estimate convolution kernels to hallucinate frame interpolation for each patch. Also, Niklaus et al. [29] and Jiang et al. [7] proposed to compute the bidirectional flow to warp the two input frames *halfway towards* each other as well as its context features to synthesize the middle frame. Similarly, Liu et al. [30] proposed a voxel flow layer given two consecutive input frames that estimates the interpolated motion vector field and an occlusion map to generate the output frame. Oh et al. [31] proposed Eulerian motion representation and its frame interpolation application as well as extrapolation. A recent work by Bao et al. [6] utilized monocular depth information (along with flow, context features, and kernel methods) to improve performance. Our work is also based on the bidirectional flow but able to extend to multi-frame input in a permutation invariant way. None of the prior arts takes into account the invariance property.

### 2.3. Spatio-Temporal Super Resolution

Although the success of deep CNNs have greatly influenced the SR and frame interpolation tasks, deep approaches for joint SR and frame interpolation have only started being explored recently, including the recent work FISR [32]. Conventional approaches, e.g., [33,34,35,36,37], remain sub-optimal due to its hand-crafted features and independent processing of spatial and temporal SR. We propose a joint SR and frame interpolation method which effectively generates the intermediate HR image.

### 2.4. Permutation Invariance

For neural networks, switching the order of the inputs generally leads to change in the output. According to [9], CNN assigns some undesirable meaning to the ordering of inputs and is difficult to *unlearn*. For order agnostic input data, this property is counterproductive. Recent works have attempted to alleviate this issue on set-valued inputs on various tasks. Ref. [11] proposed a permutation invariant networks by applying symmetric pooling layers, Ref. [38] leverage it for deep multiple instance learning, and [10] proposed to use simple commutative operations, e.g., average or max-pooling, for 3D point cloud processing. Motivated by Qi et al. [10], Aittala and Durand [9] introduced a permutation invariant method for image deblurring via burst images. Our method makes use of the principles of permutation invariant networks to address joint SR and frame interpolation.

## 3. Proposed Method

Our architecture jointly learns the appropriate features for spatial SR as well as frame interpolation at the same time. The key idea is to treat the multiple input frames equally regardless of their order. We propose the permutation invariant residual network which is able to learn complementary representations captured from the input frames that are refined through multiple layers of the constituent permutation invariant residual blocks. Then, the refined features are upscaled and fed through a final CNN decoder for high-resolution inference. We describe the network architecture in detail in Section 3.1, and explain the training scheme in Section 3.2.

### 3.1. Network Architecture

**Figure 1:**
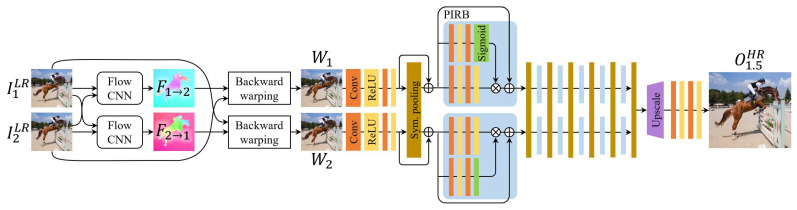
Illustration of the proposed architecture. For simplicity, we illustrate a case of two input frames but not limited. Given low-resolution image inputs $I_{1}^{LR}$ and $I_{2}^{LR}$, our architecture warps the input frames to the intermediate positions represented by $W_{1}$, $W_{2}$. These are fed through our permutation invariant module to extract complementary features. The features are upscaled and fed through a series of CNNs generating the high-resolution interpolated frame $O_{1.5}^{HR}$.

Our architecture consists of (1) the bidirectional optical flow computation and warping module, (2) the permutation invariant residual network (PIRN), and (3) the final upsampling CNN decoder. The entire network is trained end-to-end, optimizing all components to the joint SR and frame interpolation task. An overview is shown in Figure 1. For simplicity, we explain the two input frame case, but our method is not limited and can be extended to multiple frames without modification.

#### 3.1.1. Flow Estimation Module

Given two input frames $I_{1}^{LR}$ and $I_{2}^{LR}$, the flow estimation CNN estimates the bidirectional flow between them, yielding flow maps $F_{1\to 2}$ and $F_{2\to 1}$. Then, we backward-warp each frame to the intermediate position by applying half the magnitude of the flow maps, producing warped frames $W_{1}$ and $W_{2}$. The technique of using bidirectional flow provides both warped frames from each input frame to the intermediate position, which helps the network learn how to handle pixels with occlusion and disocclusion [7,12]. The warped frames are then fed through a series of convolutional layers independently and then through our permutation invariant module. For the intraframe SR without temporal interpolation, i.e., recovering $I_{1\mathrm{or}2}^{HR}$, we can seamlessly feed the backward-warped image to the target frame instead of the intermediate position.

#### 3.1.2. Permutation Invariant Residual Network (PIRN)

To extract a unified feature from both $W_{1}$ and $W_{2}$, the usual approach would be to simply concatenate and pass them through a CNN as done in [7,12,29], or use a recurrent neural network (RNN) to sequentially feed them [2]. However, both approaches are prone to permutation variance, meaning that by switching the order of the inputs will lead to changes in the output. This phenomenon is generally unfavorable for tasks agnostic to such order (e.g., SR and frame interpolation) since the learned feature most likely assigns unwanted meaning to order. Although one could argue that during training, the neural networks will learn to disregard order information, as argued by [9], this claim is theoretically unsatisfying and empirically is not the case.

By considering both images as a *set of inputs* rather than ordered inputs, it is possible to extract a complementary representation from both images as follows. Each input image is processed by a shared network, followed by a *symmetric* pooling layer, namely max-pooling or average-pooling across input members (i.e., two input image features as elements in a set). This process repeats across several layers, leading to deeper representations. The underlying idea is that through end-to-end training, the shared network will learn to extract features for which the pooling is meaningful. Intuitively, the symmetric pooling operation acts as combining features for every spatial position by considering each member equally, eventually leading to refined features accordingly. The complementary features are refined with every layer of our permutation invariant residual block (PIRB) due to the concatenation with each per-member input feature, which creates a *contrasting mechanism* for each member and the complementary feature. This helps prevent each per-member features from losing its individual information from repeated symmetric pooling. An illustration of the PIRB and PIRN are shown in Figure 1.

We also incorporate an attention mechanism to effectively attend to important salient regions and robustly handle occlusion and disocclusion present in the inputs. In addition, attention can enable canceling feature aggregations that may potentially yield unwanted artifacts. The attention mask is computed by two convolutional blocks where the last activation is the sigmoid function. This mask is applied to the output features via element-wise product, enforcing gating. Furthermore, unlike vanilla convolution operations which are spatially equivariant (identical filters are applied to every pixel), the attention module provides spatial and channel-variant attention maps to modulate local contrast. Finally, we devise the neural network block with residual learning. A PIRB encompasses the symmetric pooling and CNN as a single unit. Specifically, given a set of input features $F_{\mathrm{in}}=\left\{f_{1},f_{2}\right\}$ at a PIRB layer, we first apply symmetric-pooling sym(·) (max or average-pooling) across the channel axis of both features to compute a representative set feature $f_{\mathrm{set}}$, i.e., $f_{\mathrm{set}}=\mathrm{sym}\left(F_{\mathrm{in}}\right)$. For the branch of the *i*-th input $I_{i}$ (i={1,2}), PIRB can be expressed as:

(1) $$\;{\mathrm{PIRB}}_{i}(F_{\mathrm{in}}):=\;f_{i}\;+\;{\mathrm{Conv}}_{R}\left([f_{\mathrm{set}},f_{i}]\right)⊙{\mathrm{Conv}}_{S}\left([f_{\mathrm{set}},f_{i}]\right),$$

where ${\mathrm{Conv}}_{R}\left(\cdot \right)$ and ${\mathrm{Conv}}_{S}\left(\cdot \right)$ are convolutional blocks with the last activation as ReLU and sigmoid respectively, $f_{i}$ denotes an individual input feature member, [·] the concatenation operation, and ⊙ the element-wise multiplication. We denote the output of PIRB as the individual output feature $f_{\mathrm{out},i}={\mathrm{PIRB}}_{i}\left(F_{\mathrm{in}}\right)$. Note that $f_{\mathrm{set}}$ represents the complementary feature representation in both spatial and temporal aspects, after passing the set of inputs through the symmetric operation. Our permutation invariant layer with $f_{\mathrm{set}}$ is built on the theoretical foundation of Zaheer et al. [11] and Qi et al. [10]; thus, our design is not only empirically effective but also theoretically sound.

Extracting the complementary information from both inputs is also a key component for spatial SR. Given the warped frames $W_{1}$ and $W_{2}$, our network will learn to extract features complementing each other via different sub-pixel offset information. According to [39], MISR requires that the input contains multiple aliased images, sampled at different subpixel offsets. The different phases of low frequency is leveraged for SR. Our problem can be thought of as MISR or VSR where adjacent frames are used as information, but the key difference from our problem is on missing a reference frame (i.e., the center frame). Since there is no reference to work with, our problem is regarded as more challenging.

#### 3.1.3. Upsampling CNN Decoder

To prevent excessive memory usage, we incorporate the upscaling module only once at the final layers of the entire network. Also, our network does not incorporate any dense connections which require significant memory usage as well as the number of weight parameters. Although recent state-of-the-art makes use of the popular dense connections among multiple up/down-scaled features [1,2,5], our method shows superior results without such process. Thus, in this work, we can focus on the effects of the learned features via our proposed PIRN, but our network can be potentially improved by deploying a more advanced upsampling decoder.

#### 3.1.4. Network Architecture Details

We provide details on our full deep network in Table 1: PIRN. The layers and those parameters are shared for each input member for symmetry. Note that the PIRB modules (2nd row section of Table 1) consisting of PConvs are repeated 6 times.

### 3.2. Training Details

To train our network, we utilize subsequent frame triplets provided in high-resolution $I_{1}^{HR}$, $I_{2}^{HR}$, and $I_{3}^{HR}$, and down-sample (bicubic) them to low-resolution images. Thus, given the low-resolution images $I_{1}^{LR}$ and $I_{3}^{LR}$ as input, our model produces the interpolated high-resolution frame $O_{2}^{HR}$. We use the pixel-wise $\ell^{1}$-loss defined as $L_{1}={∥I_{2}^{HR}-O_{2}^{HR}∥}_{1}$. We also apply the perceptual loss utilizing the response from the relu4_3 layer of VGG-19 [40]: $L_{p}={∥\phi \left(I_{2}^{HR}\right)-\phi \left(O_{2}^{HR}\right)∥}_{2}^{2}$, where ϕ(·) denotes the feature vector of the relu4_3 layer. We take the sum of $L_{1}$ and $L_{p}$ as the final loss, $L_{total}=\lambda L_{1}+\mu L_{p}$, where we set λ and μ to 2.0 and 0.01 respectively.

We train our entire framework using the Vimeo90k dataset [12] of size 448×256 using the Adam optimizer with $\beta_{1}=0.9$ and $\beta_{2}=0.999$, a learning rate of 0.001, and mini-batch size of 16 samples. We utilize 51,313 training examples, and train the architecture for 100 epochs, with a linear decay in the learning rate (until reaching learning rate of $10^{-5}$) applied starting from epoch 10. To eliminate potential dataset bias, we also augment the training data on the fly by randomly reversing the frame order and applying horizontal and/or vertical flips. Our framework is implemented via PyTorch. We train our model for 2 days using two NVIDIA Titan X (Maxwell) GPUs.

Our end-to-end trainable network enables learning the appropriate features oriented to solving the joint SR and interpolation task. In the process, the flow estimation module uses the PWC-Net [41] as the backbone architecture for flow estimation. It is important to leverage the pretrained knowledge of the optical flow module. Without the initial knowledge, other network modules may suffer from learning meaningful task information, due to their random initialization. To warm-start the training process, we fix the weights of the flow estimation module for the first epoch to prevent any erroneous gradients from back-propagating to the flow module. After the first epoch, the entire network is trained end-to-end enabling the flow module to learn task-specific flow characteristics [12].

## 4. Experimental Results

Our method is applicable and tested on the sensors that are standard video cameras. Thus, we evaluate the effectiveness of our method on the following three datasets, Vimeo90k [12], Vid4 [13], and SPMCS [14]. The Vid4 dataset contains challenging videos with dynamic movement, however has a relatively small number of videos of only four. The SPMCS dataset, on the other hand, contains a large diversity of videos but relatively limited movement. The Vimeo90k dataset contains a vast variety of videos with various dynamic scenes. We compare against various combinations of state-of-the-art SR and frame interpolation methods as well as the recent competing methods. Throughout the experiments, we focus on ×4 SR factor and ×2 frame upsampling factor. The typical video data format we use as test samples typically have frame rates of 29 FPS and a video length of 1 s, and video resolutions are 960×540 (SPMCS), 448×256 (Vimeo90k), and 720×480 or 720×576 (Vid4), respectively. Note that our model is trained only on Vimeo90k, but tested on the other datasets without fine-tuning.

### 4.1. Quantitative Results

We measure PSNR (We use the scikit-image library to compute PSNR.) and SSIM which are the mainly used metrics for both SR and frame interpolation tasks. Note that our model is only trained on the Vimeo90k dataset, but is evaluated on the three datasets, i.e., assessing the challenge of *generalization*. Nonetheless, our method performs favorably for each dataset.

The comparison baselines were constructed by sequentially applying the state-of-the-art SR and frame interpolation methods. For the selected methods in frame interpolation, we include SepConv [8], SuperSlomo [7], and DAIN [6], while for SR (or VSR) methods, we include RBPN [2], and DBPN [1] as well as the bicubic method as reference. The quantitative results from combining these methods are shown in Table 2. We combined both methods by applying frame interpolation followed by the SR method, as well as in the reverse order, and report the better performing combination. Nevertheless, we found that applying frame interpolation first, then SR performed slightly better for most cases, which agrees with the findings from [32] as well (please refer to the Supplementary Material for comparisons to Kim et al. [32] ). We observe that our PSNR performance is comparable or slightly lower than the best performance while showing consistent boost in performance in terms of SSIM. Given the fact that SSIM was designed to improve traditional quality metrics such as PSNR, the SSIM results suggest that our method conveys favorable visual quality. This is rather prominent in our visual comparisons discussed in the next.

It is worth noting that our method is able to produce more visually pleasing results compared to the baselines despite having significantly fewer number of parameters. As shown in Table 2, our model contains 12.0 million parameters while the sequential methods have at least 19.8 and at most 36.8 million parameters. The baseline with the most number of parameters (DAIN-RBPN) shows the best performance among baselines, while SuperSlomo-Bicubic having the the smallest number of parameters is among the lowest performing methods. Our method outperforms this baseline with only one-third of its parameter count, in terms of visual quality. This signifies that our method can learn the complementary features learned for the joint SR and frame interpolation tasks, because without complementary feature learning, no better performance than single task models can be obtained.

Similar to the investigation by [9], we compare our method by switching the symmetric pooling layer between max pooling and average-pooling. The performances of using either operations do not show significant difference, which agrees with the investigation in [9]. However, using the average-pooling does convey slight improvement in metric performance. This may be due to the *combining* process induced by the average-pooling rather than the *selection* process induced by max-pooling which may be prone to dropping complementary information.

Furthermore, we compare with a recent work on spatio-temporal deep learning STAR [42], and the baselines included in the paper shown in Table 3.

### 4.2. Qualitative Comparisons

To demonstrate the visual advantage of our approach, we provide visual comparisons between our method and the baseline which has the *best PSNR performance* (among baselines) on each dataset. Note that the baseline with the best PSNR performance also tends to be the best SSIM performance among baselines suggesting a challenging comparison to our method.

**Figure 2:**
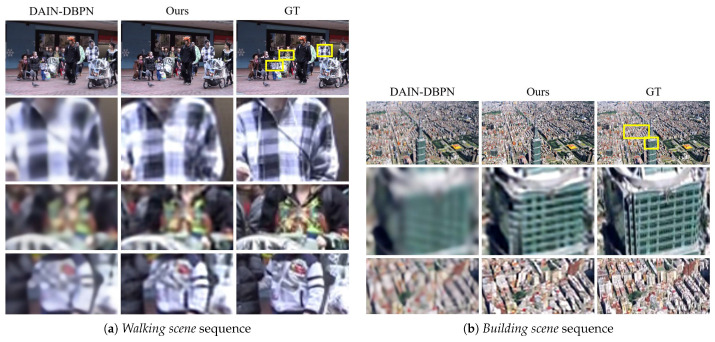
Comparison on the (**a**) Vid4 dataset and the (**b**) SPMCS dataset. Our method shows favorable preservation of (**a**) the pattern and textures on clothes, and (**b**) the pattern and textures on a building. The magnified regions are denoted as yellow boxes.

For the Vid4 dataset, we provide the comparison in visual detail between our method and DAIN-DBPN. Since PSNR is based on measuring the signal-to-noise ratio, it is rather tolerant to image blur; thus, PSNR fails to accurately assess image quality *w.r.t.* the human visual system [43]. Although DAIN-DBPN conveys image blur, the PSNR metric is generous towards it while the SSIM score is significantly lower than that of our method. From the comparison, we can observe that the details of our results are relatively more preserved than the DAIN-DBPN baseline shown in Figure 2a. In particular, our approach manages to preserve the detailed patterns on the car wheels and texture on the bushes and trees. The similar is true for the SPMCS dataset where DAIN-DBPN shows higher PSNR but lower SSIM due to blurry results as shown in Figure 2b. In particular, our results on a video frame of a cactus shows the sharp characteristics whereas the baseline conveys heavily blurred results.

**Figure 3:**
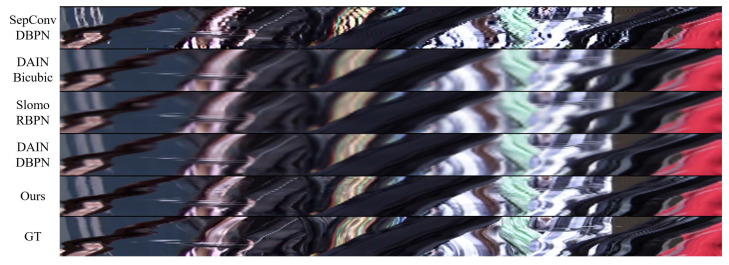
The *x*-*t* slice of the *Walk* scene in the Vid4 dataset, where *x* is the 220-th pixel row.

In Figure 3, we present the *x*-*t* slice (horizontal pixel row slices of consecutive frames, stacked beneath each slice) comparisons to convey how our results perform temporally. Our method shows sharper details closer to the ground truth.

**Figure 4:**
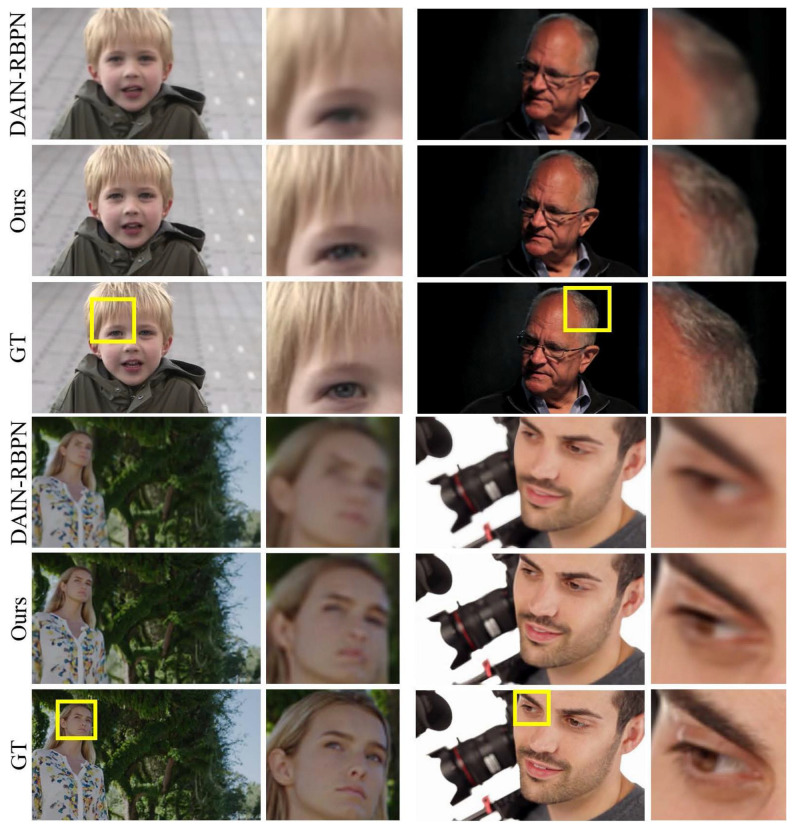
Comparison on the Vimeo90k dataset. Our method shows favorable human face reconstruction compared to DAIN-RBPN. The magnified regions are denoted as yellow boxes.

In Figure 4, we compare DAIN-RBPN and our method on the Vimeo90k dataset. Although the PSNR is comparable, the performance gap on SSIM is relatively large. The texture and facial details are blurry for DAIN-RBPN while our method shows relatively improved results. In particular, the texture of the hair, faces and the eyes is well preserved by our method. Preservation of facial attributes is an important application for SR as well as frame interpolation.

**Figure 5:**
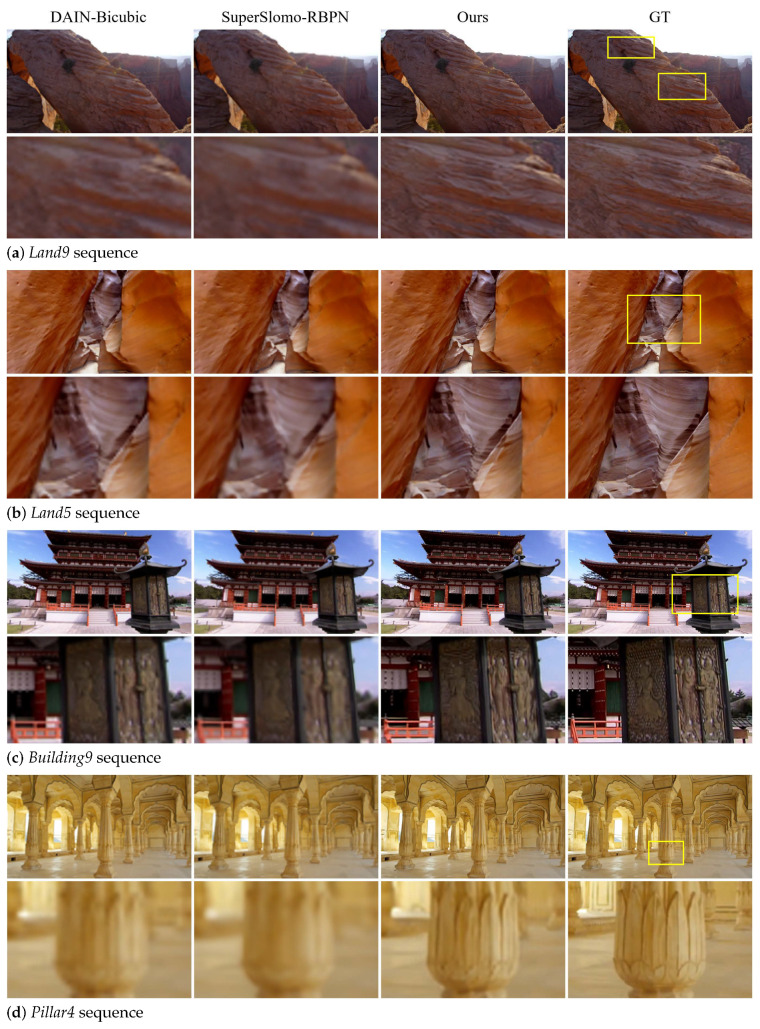
Comparison on the SPMCS dataset. We provide comparison to DAIN-Bicubic and SuperSlomo-RBPN for reference. The magnified regions are denoted as yellow boxes.

In Figure 5, other competing baselines are compared on the SPMCS dataset. The spatial video super-resolution methods and frame interpolation methods used in the combined competing methods are strong baselines in each respective field, but this result shows that combining each of the best method results in sub-optimal performance.

**Figure 6:**
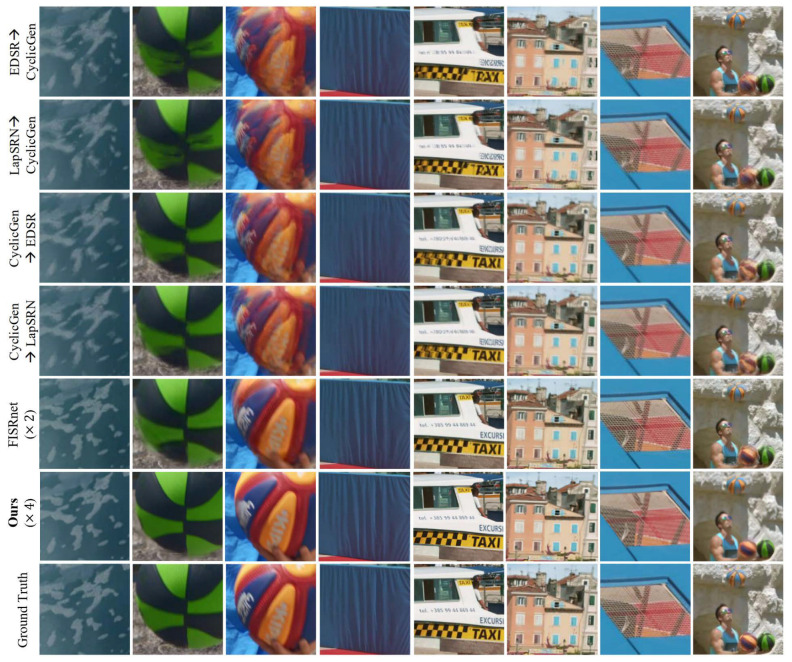
Visual comparison to FISR [32] (×2) and our method (×4) along with additional baselines. Despite the disadvantage of our method performing ×4 spatial upsampling compared to ×2 for the other methods, our approach shows even favorable visual quality if not comparable. We compare with the visual results displayed in the FISR [32] paper. The other methods show bleeding (2nd column) or ghosting artifacts (3rd column) while our method does not. Reprinted/adapted with permission from Ref. [32]. Copyright 2019, Soo Ye Kim.

We present another comparison on the 4K60fps dataset [32]. To provide visual reference to the performance on the 4K dataset provided by FISR [32], we visually compare our method with FISR and its baselines in Figure 6. Our experimental settings are different causing an unfair advantage for FISR over our method. Works on super resolution normally assess there performance on well known datasets like Vimeo90k [12] with ×4 upscaling. This is an unspoken convention for empirical evaluation so that comparison can be done on similar settings. However, FISR uses a custom dataset with ×2 upscaling whereas standard experimental procedures are mostly ×4 on major datasets such as the Vimeo90k, which our experiments are mostly based on. Another significant advantage that FISR possesses over our method and other baselines is that it is trained via the 4K60fps training set, whereas our method is trained on the Vimeo90k dataset. Nevertheless, we run our method on the FISR custom dataset without fine-tuning which is a serious handicap (×4 upscaling while baselines are ×2), but our method manages to produce sufficient results. Notice that our method is at least comparable to the performance of FISR on the 4K60fps dataset, but also conveys better details (e.g., no ghosting artifacts on basketball image on the 2nd and 3rd columns).

### 4.3. Ablation Study

To investigate whether the key components of our method contributes to the performance, we conduct an ablation study in Table 4. We compare the effects of the permutation invariance, switching the input order, and attention module. We denote these baselines as *Order dependent*, and *w/o Attention module* respectively which are compared with our *Order independent*. Furthermore, we also compare the performance of the two and four frames input cases to verify the effects of permutation invariance. Note that our *Order independent* is the full model which is the symmetric pooling layer added to the *Order dependent* baseline.

The results show that our full method comprising of the permutation invariance and attention modules perform the best, suggesting that all modules contribute to the performance of our approach. It is worth noting that the permutation invariance gives the largest boost in performance while the attention module shows slight improvements. Moreover, the *Order dependent* baseline with $\left(I_{1},I_{2}\right)$ and $\left(I_{2},I_{1}\right)$ input orders show subtle differences in performance and lower performance which indicates that the network has assigned some meaning to input order, i.e., the arrow of time [44]. This slight difference is significant to suggest that the order dependent models are potentially less stable, while the full model benefits from permutation invariance, resulting in approximately 0.2 dB boost in PSNR performance. Thus, adding the permutation invariant characteristic, namely the symmetric pooling allow symmetric handling of inputs that can potentially be more stable. The 0.2 dB gap is not negligible given that it is collapsed information, where there could be some samples that have noticeable performance variations due to the input order. Also, PSNR may not perfectly represent visual quality [43], and that the results shown in Figure 2, Figure 3, Figure 4, Figure 5 and Figure 6 show noticeable visual improvement.

### 4.4. Application: Video Compression Effect

Today we are experiencing an abundance of video data constantly being uploaded to the web. The trends in video data show that the number of videos is increasing as well as their duration. It has been prospected that videos will take up 82% of the entire Internet traffic by 2022 [45]. Moreover, video capture technology has advanced significantly, allowing higher resolution and higher frame rate videos. Due to these trends, memory consumption (especially for mobile devices), as well as transfer bandwidth (for streaming applications), have become major issues. Thus, in this paradigm, image/video enhancement techniques can be potentially exploited as complementary to conventional compression methods.

While this is a preliminary experiment, we demonstrate the benefit of our approach that produces an overarching trade-off between video size and quality against standard video codecs (H.264). For this test, we use the compressed H.264 videos spatially down-sampled by ×0.25 and temporally sub-sampled by half as input, and compare with the video directly compressed by H.264 with full resolution. We set the compressed video size to be similar (although our method is slightly smaller) by rate control, and assess the visual quality. All the used original videos are of size 960×540 with 29 FPS of frame rates and 1 s of video length.

**Figure 7:**
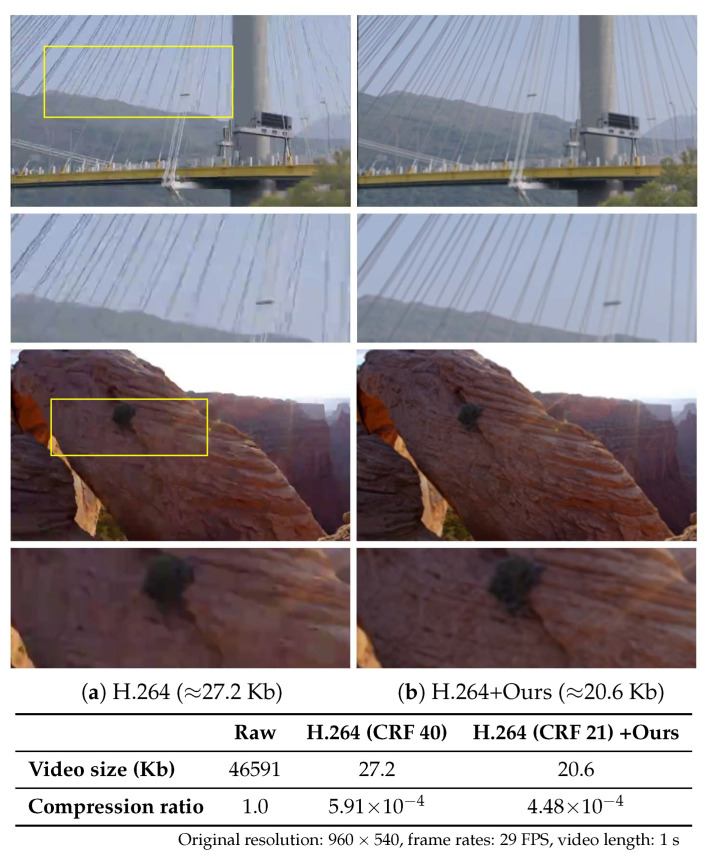
Comparison to video compression. Visual contrast between the (**a**) H.264 compressed frame and (**b**) our generated frame from H.264 compressed frame. We adjust the compressed video size to be similar by tuning the constant rate factor (CRF) to investigate the quality difference at limited bandwidth scenarios. For comparison, the H.264 reference uses CRF = 40, while CRF = 21 is used when applying our method. The magnified regions are denoted as yellow boxes.

Compared to the standard video compression, our method shows favorable visual quality as shown in Figure 7. Although our approach involved spatial SR and temporal upsampling steps, our method can reconstruct spatial and temporal information, whereas the compressed frame conveys compression artifacts. In particular, the pure H.264 compression introduces compression artifacts on the wires of a bridge, and details on a rock.

**Figure 8:**
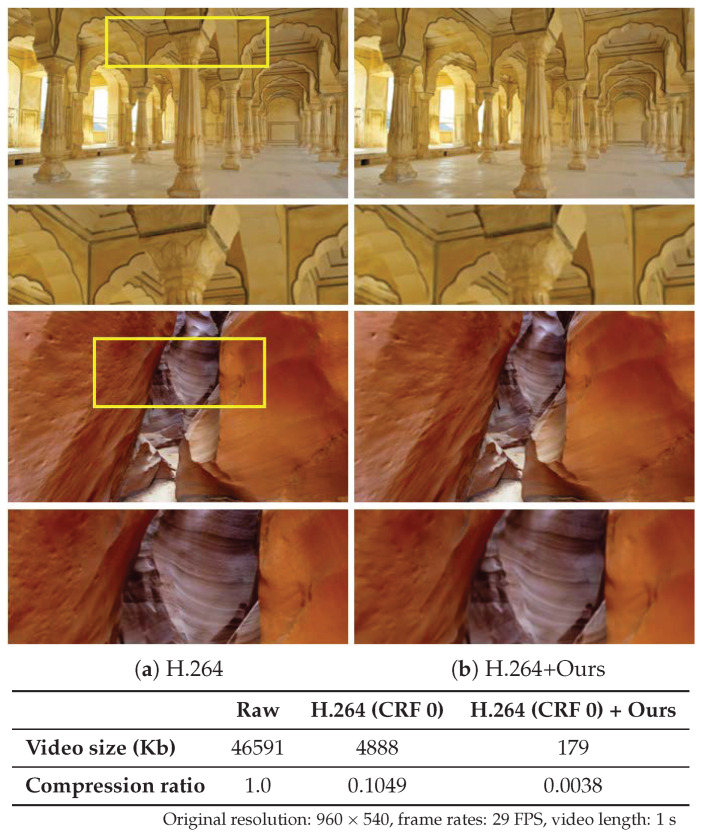
Comparison to video compression. Visual contrast between the (**a**) H.264 compressed frame and (**b**) our generated frame from H.264 compressed frame. In this experiment, different from Figure 7, we used the same CRF = 0 for comparing compression ratios. The magnified regions are denoted as yellow boxes.

We provide another comparison of our approach for additional information in terms of video size and quality against standard video codecs (H.264). For this additional experiment, we compare the video size using a lossless compression (CRF 0). Since our method involves spatial and temporal reduction in addition to compression, our results show significantly smaller size while comparable in visual quality as shown in Figure 8. Please note this is for demonstrating the potential effectiveness of the proposed method without any claim.

## 5. Discussion

Our method largely relies on the quality of the optical flow module. When the optical flow fails, the performance would be degraded. Fortunately, failures of a sparse number of optical flow pairs would not directly degrade our method. It is because our permutation invariant layer can robustly deal with such sparse outliers by virtue of selection or smoothing property of the max or average operation, respectively, In this sense, a more number of input frames would improve the robustness against the failure of optical flow.

However, increasing the number of input frames is not always available. Longer distant frames from the target frame would increase the chance to fail optical flow estimation. It means that increasing the number of distant input frames is likely to increase the chance to introduce erroneous features. If more than a majority of the features are contaminated by the failure of optical flow, then it would yield a quality drop. Nonetheless, our method can be easily improved if we replace the optical flow module with a more advanced state-of-the-art optical flow method, e.g., [46,47].

## 6. Conclusions

We propose a joint SR and frame interpolation method of videos. We devise a permutation invariant block that enables to learn complementary features beneficial for both tasks. We demonstrate that our method shows favorable performance against the competing methods and baselines consisting of the state-of-the-art methods despite a smaller number of parameters. Since our method is able to enhance both spatial and temporal information from a compressed form, our work can be used to deal with limited storage memory or bandwidth, which have practical values such as video streaming.

## Figures and Tables

**Table 1:** Permutation invariant residual network (PIRN) details.

Layer Name	Filter Size	Channels	Stride	Upscale	Activation
Conv0	3×3	3	1	-	ReLU
Conv1	3×3	64	1	-	ReLU
Sym. pooling	1×1	64	1	-	Max/Avg
Concat(w/Conv1)	-	64 + 64	-	-	-
PConv_R0	3×3	64	1	-	ReLU
PConv_R1	3×3	64	1	-	ReLU
PConv_S0	3×3	64	1	-	ReLU
PConv_S1	3×3	64	1	-	Sigmoid
Sym. pooling	1×1	64	1	-	Max/Avg
Conv3	3×3	64	1	-	ReLU
UpScale	-	64	-	×4	-
Conv4	3×3	64	1	-	ReLU
Conv5	3×3	3	1	-	ReLU

**Table 2:** Spatio-temporal SR performance on the Vimeo90k, Vid4, and SPMCS datasets against combinations of the state-of-the-art interpolation and SR methods. The best performing and runner-up methods are marked in **red** and blue, respectively.

Dataset	#param.	Vimeo90k		Vid4		SPMCS	
Metric	(Million)	PSNR	SSIM	PSNR	SSIM	PSNR	SSIM
SepConv-$L_{f}$ [8]→ Bicubic	21.6	33.1487	0.9589	30.0614	0.8760	31.0992	0.9174
SepConv-$L_{1}$ [8]→ RBPN [2]	34.4	32.4599	0.9283	29.5295	0.8224	31.2464	0.9034
SepConv-$L_{1}$ [8]→ DBPN [1]	32.0	32.6833	0.9349	29.7292	0.8337	31.2743	0.9043
SuperSlomo [7] → Bicubic	19.8	32.6034	0.9556	29.7232	0.8627	30.9245	0.9115
SuperSlomo [7] → RBPN [2]	32.6	32.9948	0.9612	29.8192	0.8711	31.0364	0.9152
SuperSlomo [7] → DBPN [1]	30.2	32.9835	0.9612	29.8260	0.8710	31.0405	0.9152
DAIN [6] → Bicubic	24.0	33.0474	0.9628	30.0717	0.8931	31.0960	0.9167
DAIN [6] → RBPN [2]	36.8	33.8300	0.9730	30.4270	**0.9201**	31.2514	0.9024
DAIN [6] → DBPN [1]	34.4	33.7916	0.9737	30.4284	0.9196	**31.2758**	0.9029
Ours (Max-pooling)	12.0	34.3556	0.9730	30.6366	0.9117	31.2392	**0.9192**
Ours (Avg.-pooling)	12.0	**34.4841**	**0.9739**	**30.7144**	0.9169	31.2145	0.9172

**Table 3:** Comparison with STAR [42].

Dataset	Vimeo90k
Metric	SSIM
TOFlow [12] → DBPN [1]	0.897
DBPN [1] → DAIN [6]	0.918
STAR-$L_{f}$ [42]	0.926
STAR-ST-$L_{f}$ [42]	0.927
STAR-ST-$L_{r}$ [42]	0.927
Ours	**0.974**

**Table 4:** Ablation study of our method including the full versions, without the permutation invariance module (in-order and reverse-order inputs), and attention module. The best performing method is indicated in bold. Please note that the number of parameters between the *Order independent* full method and *Order dependent* baselines do not differ. Further, with four frames input, the performance increases (denoted in red) as opposed to degradation, showing the effects of permutation invariance.

Dataset	Vimeo90k		Vid4		SPMCS	
Metric	PSNR	SSIM	PSNR	SSIM	PSNR	SSIM
Order independent (Avg)	34.4841	0.9739	**30.7144**	**0.9169**	31.2145	0.9172
Order dependent ($I_{1}$,$I_{2}$)	34.2758	0.9721	30.5972	0.9100	31.2385	0.9192
Order dependent ($I_{2}$,$I_{1}$)	34.2761	0.9721	30.5945	0.9099	31.2386	0.9192
w/o Attention module	34.3954	0.9732	30.6194	0.9125	31.1806	0.9165
Order independent (Max)	34.3556	0.9730	30.6366	0.9117	31.2392	0.9192
Order independent (4-frame)	**34.7363**	**0.9746**	30.5990	0.9154	**31.2914**	**0.9198**

## Data Availability

Not applicable.

## Supplementary Materials

The following supporting information can be downloaded at: https://www.mdpi.com/article/10.3390/s23052529/s1, Video S1: Qualitative comparison.
